# Recovery of Physiological Traits in Saplings of Invasive *Bischofia* Tree Compared with Three Species Native to the Bonin Islands under Successive Drought and Irrigation Cycles

**DOI:** 10.1371/journal.pone.0135117

**Published:** 2015-08-20

**Authors:** Kenichi Yazaki, Katsushi Kuroda, Takashi Nakano, Mitsutoshi Kitao, Hiroyuki Tobita, Mayumi Y. Ogasa, Atsushi Ishida

**Affiliations:** 1 Department of Plant Ecology, Forestry and Forest Products Research Institute (FFPRI), Tsukuba, Ibaraki, 305-8687, Japan; 2 Department of Wood Properties, Forestry and Forest Products Research Institute (FFPRI), Tsukuba, Ibaraki, 305-8687, Japan; 3 Division of Natural Environmental Sciences, Mount Fuji Research Institute, Yamanashi, 403-0005, Japan; 4 Center for Ecological Research, Kyoto University, Otsu, Shiga, 520-2113, Japan; INRA - University of Bordeaux, FRANCE

## Abstract

Partial leaf shedding induced by hydraulic failure under prolonged drought can prevent excess water consumption, resulting in delayed recovery of carbon productivity following rainfall. To understand the manner of water use of invasive species in oceanic island forests under a fluctuating water regime, leaf shedding, multiple physiological traits, and the progress of embolism in the stem xylem under repeated drought-irrigation cycles were examined in the potted saplings of an invasive species, *Bischofia javanica* Blume, and three endemic native species, *Schima mertensiana* (Sieb. Et Zucc,) Koitz., *Hibiscus glaber* Matsum, and *Distylium lepidotum* Nakai, from the Bonin Islands, Japan. The progress of xylem embolism was observed by cryo-scanning electron microscopy. The samples exhibited different processes of water saving and drought tolerance based on the different combinations of partial leaf shedding involved in embolized conduits following repeated de-rehydration. Predawn leaf water potential largely decreased with each successive drought-irrigation cycle for all tree species, except for *B*. *javanica*. *B*. *javanica* shed leaves conspicuously under drought and showed responsive stomatal conductance to VPD, which contributed to recover leaf gas exchange in the remaining leaves, following a restored water supply. In contrast, native tree species did not completely recover photosynthetic rates during the repeated drought-irrigation cycles. *H*. *glaber* and *D*. *lepidotum* preserved water in vessels and adjusted leaf osmotic rates but did not actively shed leaves. *S*. *mertensiana* exhibited partial leaf shedding during the first cycle with an osmotic adjustment, but they showed less responsive stomatal conductance to VPD. Our data indicate that invasive *B*. *javanica* saplings can effectively use water supplied suddenly under drought conditions. We predict that fluctuating precipitation in the future may change tree distributions even in mesic or moist sites in the Bonin Islands.

## Introduction

The Bonin Islands are the oceanic subtropical islands, which are located at approximately 1000 km south of Tokyo, Japan. Because of the rich and unique endemic flora, the islands are authorized as a World Natural Heritage site since 2011. Particular characteristics are the endemic dry dwarf forests, which develop in mountain ridges with shallow soil. Nevertheless, many native species in the islands are threatened by an anthropogenically introduced tree species, *Bischofia javanica* Blume (Pyllanthaceae). *B*. *javanica* trees vigorously spread at moist/mesic sites with relatively thick soil, especially after disturbance caused by typhoons. Their saplings have a high acclimation capacity to the fluctuating light [1] and soil nutrients [2], resulting in well adaptation to changing environments. Woody plants on islands must adapt to fluctuating soil water because of limited water resources and the frequent occurrence of typhoons (see *Study site* in Materials and Methods). Ongoing changes in precipitation and increase in frequency and intensity of typhoons can have irreversible effects on growth, forest dynamics, and forest successions, including displacement by invasive trees on the island.

Drought-induced hydraulic failure largely reduces carbon gain, resulting in dieback and even death of plant individuals [3–8]. Plants have to close the stomata to avoid excessive negative pressure in xylem water column resulting in xylem cavitation/embolism. Some woody plants detach their distal organs, such as canopy leaves, to mitigate water deficits during prolonged drought. Although leaf shedding can reduce the transpiration area of whole plants, the time lag between subsequent precipitation and the formation of new leaves results in reduced plant productivity [9]. Conversely, woody plants that retain canopy leaves under prolonged drought risk xylem cavitation. Because evergreen trees have a longer period of leaf lifespan than drought-deciduous trees with a shorter period, they tend to have higher resistance to drought-induced cavitation accompanied with narrower vessels and dense wood [10–14], consequently, they may have a low productivity via a low hydraulic efficiency and high construction costs of wood [15–18].

Circumventing xylem cavitation in Bonin Islands species may be important to maintain their productivity under fluctuating water conditions caused by prolonged drought and rainfall [19]. Wood density is a key function as an index of efficiency and stability in hydraulic function and drought tolerance [20,21]. However, the hydraulic vulnerability of xylem is determined by the hydraulic architecture and anatomical structure of xylem, such as pit dimension [11,22], vessel distribution [23], and fibre and parenchyma orientation [24]. Thus, tolerance to drought-induced cavitation can differ from what is predicted based on wood density [7,25]. Recent improvements in observation techniques, such as magnetic resonance imaging (MRI) and high resolution computed tomography (HRCT), have provided new insights into the water dynamics of intact plants [26–28]. In particular, HRCT has been improved greatly to visualize the cavitation and refilling processes in intact plants at the cellular level [27,28]. Although there is still a possibility of artificial cavitation caused by freezing segments without releasing xylem tension [29], the use of cryo-SEM provides us the advantage of allowing us to determine which specific cells are cavitated or refilled at the individual cellular level at higher resolution [13,19,25,30–32].

In the present study, we hypothesized that resistance against xylem cavitation, the shedding of canopy leaves, and recovery of physiological traits under fluctuating soil water contents are major determinants of the interspecific variations in water-use strategy and drought tolerance. Here, we compared these processes in the potted saplings of an invasive and three native tree species in the Bonin Islands under repeated drought-irrigation. Cavitation processes were examined at the cellular level using cryo-SEM microscopy. In this study, we demonstrate that fluctuating precipitation in the future will be an important factor for causing changes in tree distribution and forest structure even at mesic or moist sites.

## Materials and Methods

### Study site

In the Bonin Islands (Ogasawara Islands in Japanese; 26°39′N, 142°09′E; highest altitude is 326 m ASL), the annual precipitation is approximately 1200 mm, and frequent dry periods lasting 10 to 15 days occasionally occur, especially in summer. From 2000 to 2010, the mean annual temperature was 23.1°C and the mean maximum temperature in summer (June to October) was 29.5°C (Chichijima Meteorological Observatory, the Meteorological Agency of Japan). The examined tree species are commonly found and non-protected species. We did not need permission to collect seeds of the invasive *Bischofia*. From the native trees, we collected seeds within the class II zone (no core-protected sites) of the National Park (27°3′37″N, 142°13′19″E) and received permission before collecting seeds from the Ministry of the Environment in Japan.

### Plant materials

Four predominant tree species, *Distylium lepidotum* Nakai (Hamamelidaceae), *Hibiscus glaber* Matsum. (Malvaceae), *Schima mertensiana* (Sieb. Et Zucc.) Koidz. (Theaceae), and *Bischofia javanica* Blume (Pyllanthaceae), were selected in this study. These species were found to grow in various sites in the Bonin Islands, ranging from dry ridges to wet valleys. *D*. *lepidotum*, *H*. *glaber*, and *S mertensiana* are endemic to the islands. *D*. *lepidotum* (<7 m high) is commonly found along the ridges of mountains with shallow soil, whereas *S*. *mertensiana* (<13 m high) and *H*. *glaber* (<16 m high) tend to grow at sites with various soil depth from the ridge to valley [33,34]. *B*. *javanica* is an invasive species introduced to the islands in the early 1900s. Their distribution is expanding into mesic and moist forests and the top canopy reaches 20 m high. *B*. *javanica* is an evergreen species, in which leaf lifespan is 1.4 year on the island [35]. The seedlings of *B*. *javanica* can grow rapidly under changing light conditions, following disturbances caused by typhoons, compared with those of native tree species [1,36]. Seeds were collected from Chichi-jima Island and were germinated on a vermiculite tray in a naturally illuminated phytotron chamber at the Forestry and Forest Products Institute in Tsukuba, Ibaraki, Japan. When the seedlings were approximately 50 mm high, each individual was transplanted into a 1.8-l plastic pot containing porous clay granules (Hydroculture; Takamura Ltd., Japan). The saplings were grown under controlled temperatures (28°C during the day and 23°C overnight) and relative humidity (60% during the day and 70% overnight). They were also fully irrigated and fertilized with liquid fertilizer, 0.5 l of 1000x Hyponex solution (22 mg Nl^−1^, 36 mg PO_4_^3−^l^−1^ and 18 mg Kl^−1^; 6:10:5, N/P/K; HYPONeX JAPAN CORP., LTD, Osaka, Japan) weekly for five to six months until the onset of experiments. When the experiments began, the average heights (mean ± 1 S.D.) were 28.2 ± 10.3 cm for *D*. *lepidotum*, were 16.6 ± 2.7 cm for *H*. *glaber*, 16.2 ± 3.5 cm for *S*. *mertensiana* and 25.7 ± 2.8 cm for *B*. *javanica*. The average stem diameters near the ground base (mean ± 1 S.D.) were 3.79 ± 1.24 mm for *D*. *lepidotum*, 6.51 ± 0.67 mm for *H*. *glaber*, 3.46 ± 0.60 mm for *S*. *mertensiana* and 7.15 ± 0.49 mm for *B*. *javanica*.

### Drought and irrigation cycles

A naturally illuminated phytotron chamber was used for the cyclic drought and irrigation treatments (i.e., drought-irrigation cycle; [19]). Potted seedlings were fully irrigated just before the onset of experiments. After the first drought period was initiated, the irrigation was ceased for 15 full days and the relative air humidity in the chamber decreased to 40% during the day and 50% overnight to expose the saplings to severe drought conditions. At 15:00 on the 15th day of the first drought period, the saplings were given 0.5 l of 1000x Hyponex solution. After irrigation, the relative humidity was immediately increased to 60% during the day and 70% overnight for two days to end the severe drought. This period was called the first recovery period. We repeated the drought-irrigation cycle twice (i.e., three cycles in total) in the controlled environment. All saplings were positioned so as not to shade each other, and were rotated weekly to minimize the effect of positioning. Four or five saplings were randomly selected for non-destructive measurements of the number of leaves and the response of stomatal conductance to the leaf-to-air vapor pressure deficit (leaf-to-air VPD). Subsets of three to six saplings were used to determine the leaf gas exchange rate and were destructively sampled to determine leaf nitrogen (N) contents and leaf water potential and for observation using cryo-scanning electron microscope (cryo-SEM). A subset of saplings was sampled every 15th day (at the end of the drought period) and 17th day (at the end of the recovery period) during each drought-irrigation cycle. On the final day of the third drought-irrigation cycle (52nd day of the experimental treatment), we sampled all saplings immediately after enumerating leaves and determining leaf physiology. As a control, four or five saplings of each species were grown under fully irrigated conditions with relative humidity of 60% during the day and 70% overnight in another phytotron chamber. In total, 35 to 46 saplings in each species were used for this experiment.

### Leaf turnover and gas exchange

The numbers of leaves of all species were counted at the onset of experiments. This measurement was also conducted in the middle (5th to 8th days) and end (13th to 15th days) of the drought period in each drought-irrigation cycle. The leaf gas exchange rates (net assimilation rate, *A*; transpiration rate, *E*; and water vapor stomatal conductance per leaf area, *gs*) were measured on the second day of each recovery period in a mature (but not senescent) leaf of each sapling with a portable open gas exchange system (LI-6400; Li-Cor Inc., Lincoln, NE, USA). All measurements were conducted under 1500 μmol m^−2^ s^−1^ photosynthetic photon flux (PPF) with red-blue LED lamps, at 25 to 30°C in leaf temeperature, and 370 μmol mol^−1^ CO_2_ concentration in the inlet gas stream in the leaf chamber of LI-6400. To avoid the midday depression in leaf gas exchange, the determination of daily maximum values was conducted in the morning and the stomatal response to leaf-to-air VPD was measured on the second day of each recovery period. During the measurements, leaf-to-air VPD in the leaf chamber was adjusted to 1.5, 2.0, 2.5, and 3.0 kPa, by gradually increasing the volume of dry air and by adding Drierite (97% CaSO_4_, 3% CaCl_2_) to the inlet gas stream. Because *gs* exponentially decreased with leaf-to-air VPD, analysis of stomatal sensitivity followed Oren et al. [37]:

$$g_{s}\;=\;-m\text{ ln }(\text{leaf}-\text{to}-\text{air VPD})\;+\text{ b}$$ (1)

where *m* is stomatal sensitivity (mmol m^−2^ s^−1^ ln (kPa)^−1^) and *b*, the reference stomatal conductance when leaf-to-air VPD equals 1 kPa. The value of *m* was determined with extrapolation and curve fitting using least-square regression analysis.

### Leaf water potential and nitrogen contents

Three leaves per individual sapling were used to examine the leaf water status. The leaf water potential at predawn (ψ_pre_) and at midday (ψ_day_) was determined with a Scholander-type pressure chamber (Soilmoisture Equipment, Santa Barbara, CA, USA). The values of ψ_day_ were measured in the leaves used for gas exchange measurement, and those of ψ_pre_ were measured in the leaves next to the leaves used to determine ψ_day_. The measurements of ψ_pre_ and ψ_day_ were conducted at the onset of cyclic treatment (as controls), and then ψ_pre_ and ψ_day_ were again measured on the final day of each drought period and the second day of each recovery period, respectively. In addition, the measurement of ψ_pre_ was conducted midway through the first drought period (on 5 to 7 days from the onset of experiments). After the measurement of ψ_day_, another leaf was sampled to measure leaf osmotic potential at full turgor (ψ_o_). To measure ψ_o_, the cut end of the petiole was immediately placed in distilled water and re-cut under water. After being stored overnight, the leaves were wrapped in a polyvinyl chloride film to prevent any water loss, stored at −80°C for at least two days, and then thawed at 20°C to equilibrate with air temperature. Leaves were divided into two parts, half of which was used to measure ψ_o_. Using an extract from the thawed leaves, the values of ψ_o_ were determined with an osmometer (Model 5520; Wescor Inc., Logan, UT, USA).

The other half was used to measure nitrogen (N) contents within the lamina. Leaf discs were cut, avoiding major veins, with a cork borer. After drying at 70°C for 72 hours, N contents was measured with an NC analyzer (Sumigraph NC-800; Sumitomo Chemical Co. Ltd., Osaka, Japan).

### Cavitation analysis by cryo-SEM observation

To visualize water distribution in the xylem conduits of the main sapling stems, cryo-SEM observations were conducted at the start and recovery period of the first drought-recovery cycle [13,19,25,30,31]. Three or four saplings that were used for leaf gas exchange rate and water potential measurement in the first drought-irrigation cycle were used for cryo-SEM observation. Because of the limited sample number, we conducted sampling for cryo-SEM observation during the first cycle only. After measuring leaf gas exchange and ψ_day_, the saplings were stored overnight in a plastic bag to equilibrate xylem water potential to water potential in the pot soil. Polyethylene collars (approximately 10 cm in length) were attached at the mid-height of the stems in the living saplings. Liquid nitrogen was poured into the collars *in situ* for 1 minute to freeze stems and sap. Because the diameter of samples was up to approximately 0.7 cm, the period of immersion time of liquid nitrogen was sufficient to completely freeze the water in the xylem conduits. Because of limited number of saplings for following physiological measurements, sampling for the observation of water dynamics was conducted only in first drought-recovery cycle. The frozen stems were then cut into 4 to 5 cm sections and stored in a deep freezer at −80°C until cryo-SEM observation. The transverse face of each stem was cleanly cut with a cryostat-microtome (HM505E, Microm, Walldorf, Germany) attached to a specimen holder. Subsequently, the specimens were transferred to the cold stage of the cryo-SEM system (JSM-6310 attached cryo-SEM unit, Joel Co. Ltd., Tokyo, Japan). After being subjected to etching at –90°C for approximately 3 minutes, the specimens were coated with gold at approximately −100°C in an evaporation unit installed next to the cryo-SEM system. The specimens were then transferred to the sample stage (at −160°C) and observed under an accelerating voltage of 3 kV. To analyze the degree of cavitation in transverse xylem, the region of interest (ROI) in each cryo-SEM image was selected along at least ten radial files of cells. Each cell had developed from cambium by periclinal division, indicating that the cells gradually developed different traits with a radial file ontogenetically, which meant the ROI was triangular-shaped. We assumed that the vessel containing the cavity at the cross surface was hydraulically dysfunctional. We then counted each of the filled and cavitated vessels, using image analysis software (Image J, National Institutes of Health, Bethesda, Maryland, USA, http://imagej.nih.gov/ij/, 1997–2011). To evaluate the differences in cavitation tolerance of each vessel among the species, we investigated the percentage of the number of cavitated vessels, instead of the percentage of sum of the transverse area of cavitated vessel.

Cochard et al. [29] suggest the possibility of artificial cavitation caused by the freezing of xylem sap under negative tension. In addition, Wheeler et al. [38] discussed the possibility of a xylem embolism caused by an experimental artifact when stem segments are cut under higher xylem tension. Therefore, we sampled fully irrigated (i.e., ψ_pre_ were close to 0 MPa) saplings for cryo-SEM observations at the start of the experiment and during the first recovery period.

### Statistical analysis

Significant differences in means among species and among measurement days were tested using two-way analysis of variance (ANOVA) and Scheffe’s post-hoc test. To compare the changes in the ratio of the number of cavitated conduits to those of filled conduits, we created a generalized linear model (GLM) with a binomial error distribution and a log-link function. The independent variables were water regime, species, and their interaction and the dependent variable was the ratio of the number of cavitated conduits to that of filled conduits. Analysis of deviance was conducted to detect differences in the independent variables in the model. Significance level was fixed at *P* = 0.05. All statistical analyses were conducted using freeware “R” version 2.1.12 with the base package (R Development Core Team, R Foundation for Statistical Computing, Vienna, Austria; http://www.R-project.org).

## Results

### Leaf turnover

**Fig 1:**
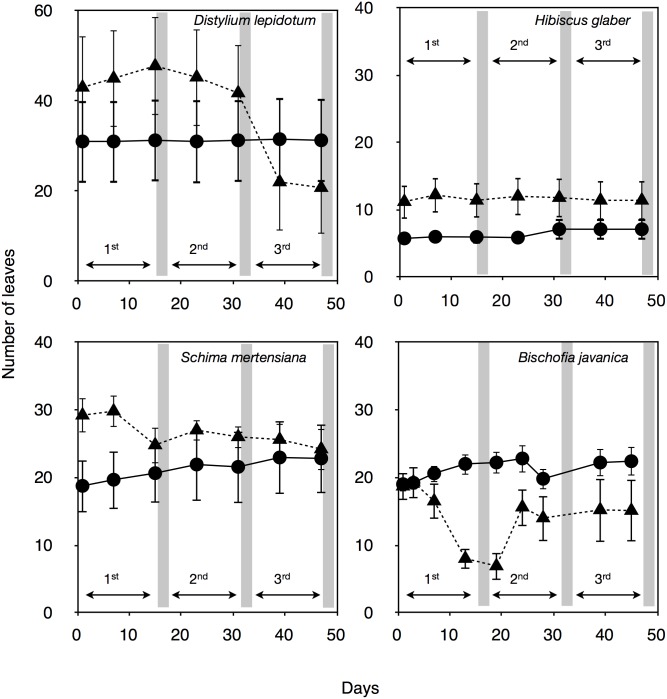
Time courses in the number of leaves under repeated drought-irrigation cycles. The control saplings (●) and the saplings in drought-irrigation cycles (▲) are displayed. Gray bars indicate the timing of irrigation following the lasting drought periods. Bars indicate ± 1 standard error of mean (n = 4–5).

The number of leaves did not change during the first and second drought cycles for *D*. *lepidotum* and *H*. *blaber* (Fig 1); however, some leaves of *D*. *lepidotum* withered and died midway in the third drought-irrigation cycle. Their dead leaves were attached to branches, indicating that no abscission layer had developed. In contrast, *B*. *javanica* and *S*. *mertensiana* shed leaves with abscission layers during the first drought-irrigation cycle (Fig 1). Furthermore, some saplings of *B*. *javanica* sprouted new leaves during the second drought-irrigation cycle under conditions where the soil water potential remained high.

### Cavitated vessels and Cryo-SEM observation

**Fig 2:**
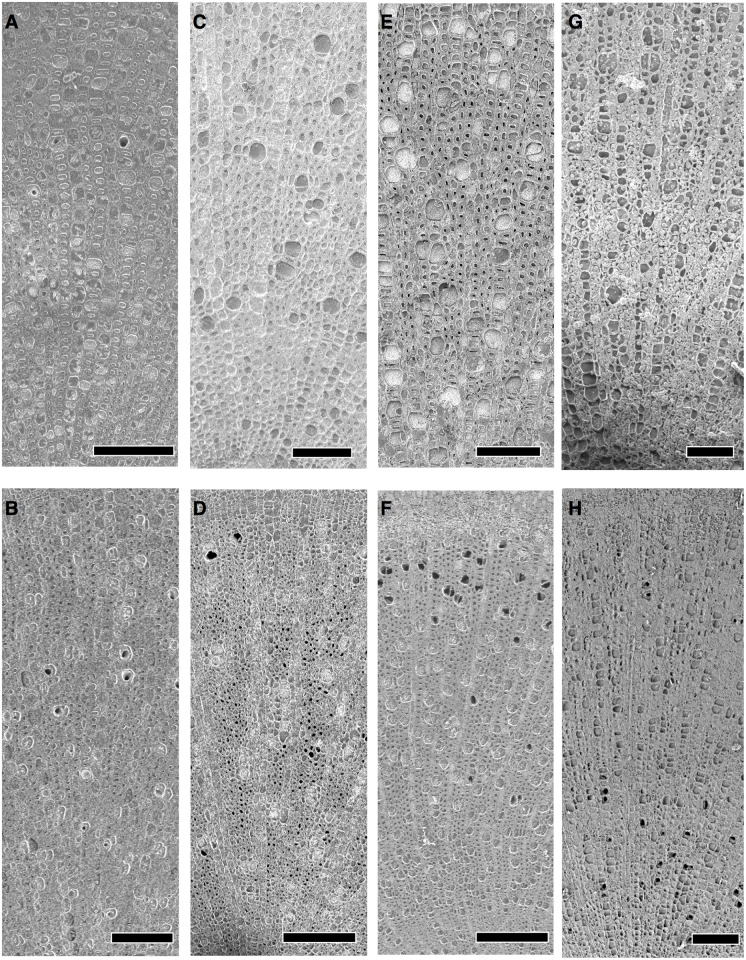
Water distribution on the transverse surface of the stems. Cryo-SEM images of the transverse surfaces of the stems, (upper panels) taken on the first day of the first drought treatment and those (lower panels) taken on the second day of the recovery (irrigation) period, in *D*. *lepidotum* (A, B), *H*. *glaber* (C, D), *S*. *mertensiana* (E, F), and *B*. *javanica* (G, H). Vessels with dark lumen indicate cavitated and vessels with light gray lumen indicate being filled with water. Note that each sapling had a nearly zero water potential when it was frozen in liquid nitrogen. Scale bars indicate 200μm.

**Fig 3:**
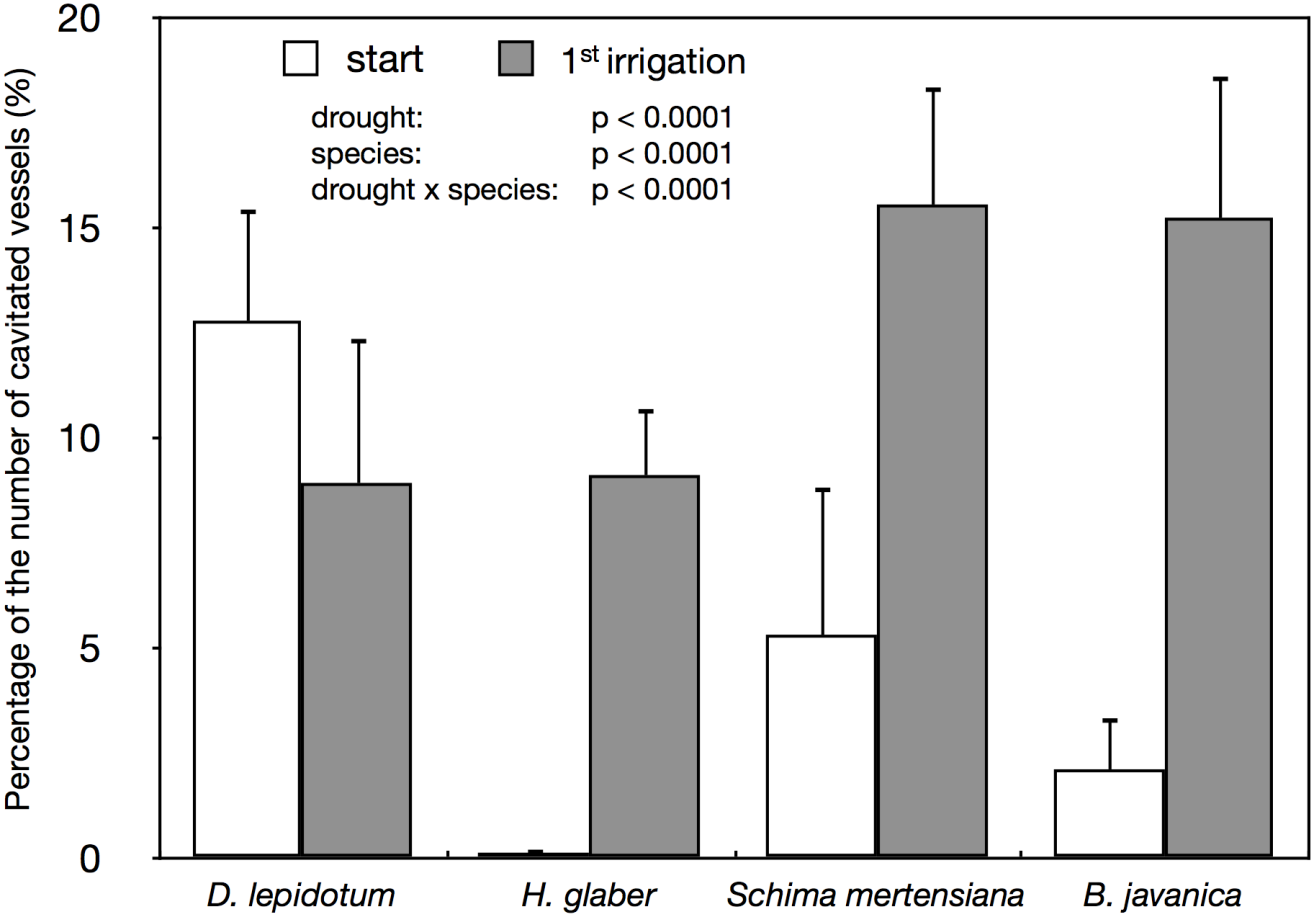
Changes in the percentage of the number of cavitated vessles. The percentage of the number of cavitated vessels at the start of the experiment and the first irrigation period. Sampling (Start) was conducted just before stopping irrigation, and sampling (first irrigation) was conducted during the recovery period of each drought-irrigation cycle. Bars indicate ± 1 standard error of the mean (n = 3–4). The p-value derived from the analysis of deviance to validate the independent variables is shown.

**Fig 4:**
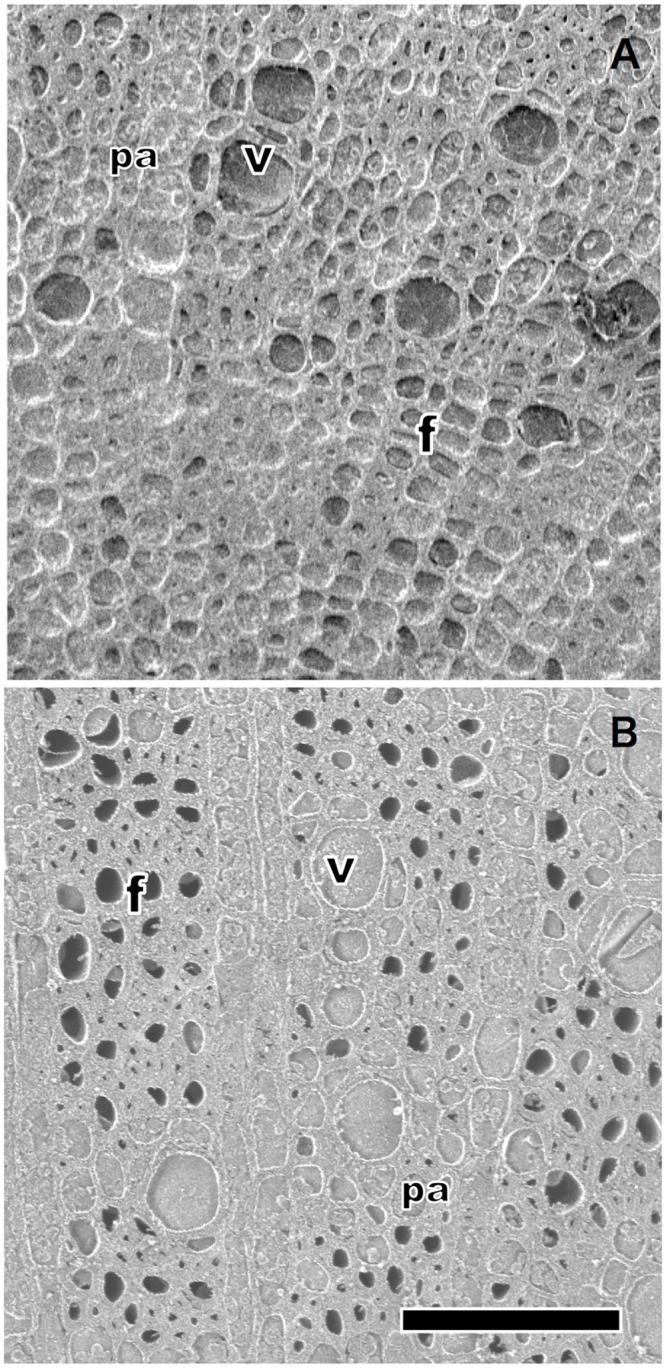
Magnified images of changes in water distribution in *H*. *glaber* during the first drought-irrigation cycle. Cryo-SEM image of the transverse surfaces of the stems in *H*. *glaber*, taken on the first day of drought treatment (A) and that on the second day of the recovery (irrigation) period (B). All vessels (v) and fibers (f) are filled with water (A). Many fibers that experienced drought were empty and large vessels conserved water (B). Cells with contents and thinner cell walls are axial parenchyma cells (Pa). Scale bar indicates 100 μm.

Cryo-SEM observation during the first drought period indicated significant interspecific variations regarding xylem hydraulics in stems (Figs 2 and 3). Native *H*. *glaber*, *S*. *mertensiana*, and invasive *B*. *javanica* showed significant progression of cavitation (Figs 2C–2H and 3). *S*. *mertensiana* and *B*. *javanica*, which shed leaves under prolonged drought, exhibited conspicuous progression of cavitation in their vessels (Fig 2F and 2H). Cavitation occurred in some vessels near pith of the invasive *B*. *javanica* (Fig 2G). All vessels and fibers in *H*. *glaber* held water at the start of the drought period (Fig 4A), but conspicuous cavitation occurred mainly in fibers (Fig 4B). Most vessels in *D*. *lepidotum* effectively held water during the first drought-recovery cycle, although they had exhibited some degree of cavitation at the start of the experiment (Figs 2A, 2B and 3).

### Leaf water potential

**Fig 5:**
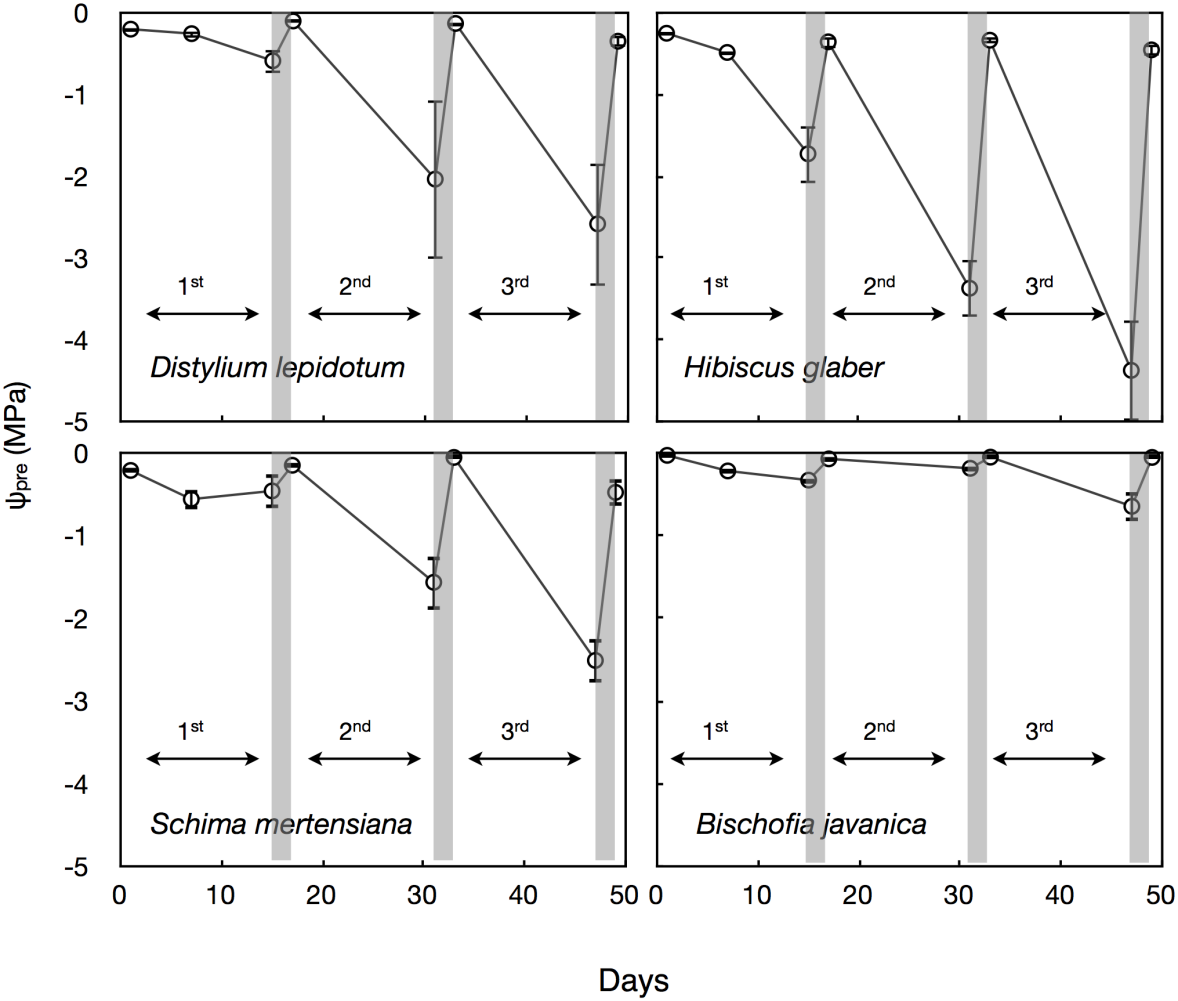
Time courses in leaf water potential at predawn under the drought-irrigation cycles. Gray bars indicate the timing of irrigation treatment following the lasting drought periods. Bars indicate ± 1 standard error of mean (n = 3–6, but n = 2 in the 3rd cycle in *Hibiscus* and in the 2nd cycle in *Bischofia*).

**Fig 6:**
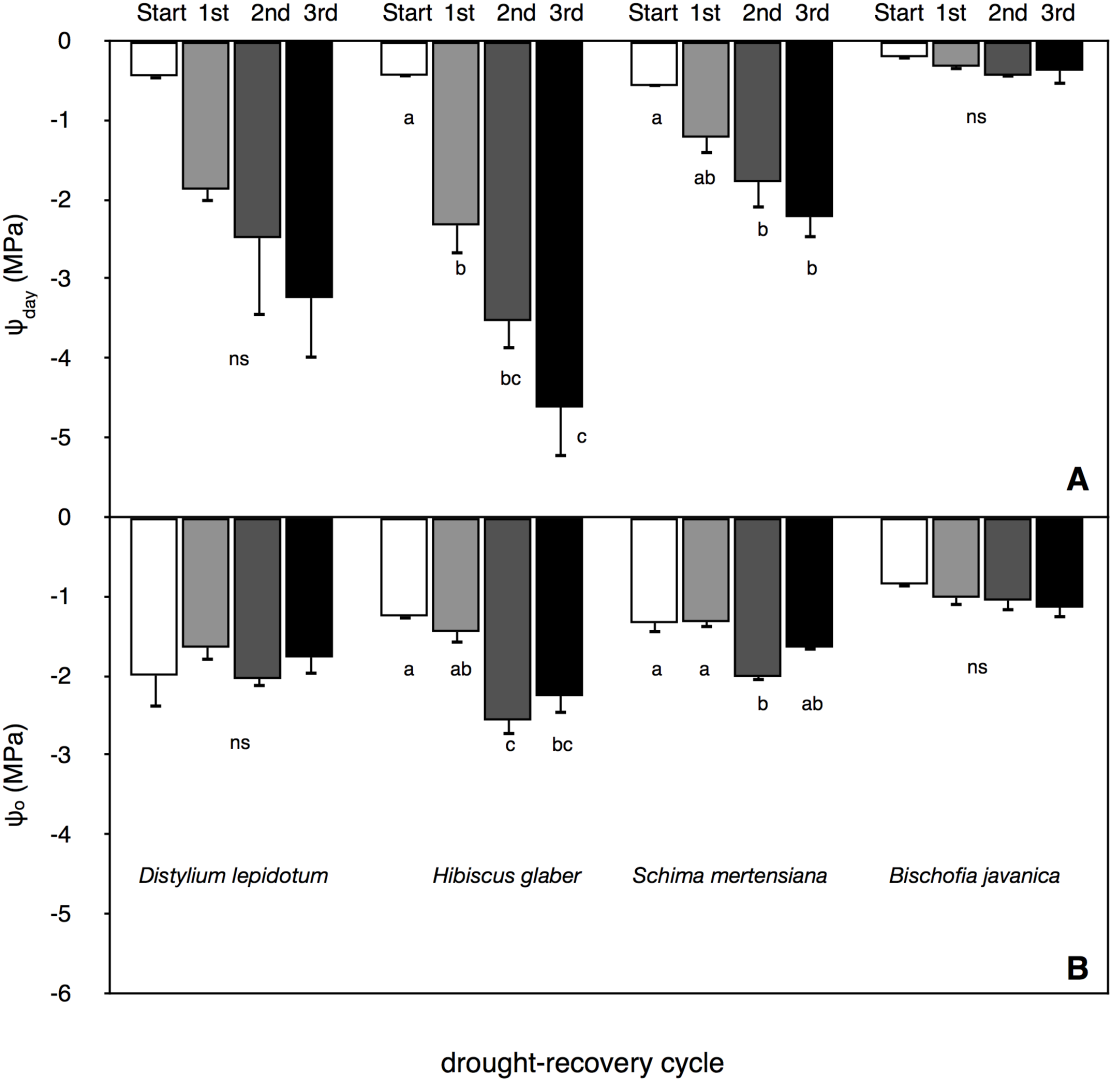
Water potential at midday and osmotic potential on the last day of each drought periods. The values of (A) leaf water potential at midday, (B) leaf osmotic water potential at full turgor measured on the last day of each drought period. The measurement (Start) was conducted just before the cessation of irrigation and the measurements (1^st^ to 3^rd^) were conducted on the last day of the lasting drought period in each drought-irrigation cycle. Bars indicate ± 1 standard error of mean. Different letters showed significant differences in each tree species (ns: no significant differences in each species). See the legend of Fig 1. in the numbers of samples.

Predawn leaf water potential (ψ_pre_) largely decreased with progressive drought-irrigation cycles in all species, except for *B*. *javanica* (Fig 5). The drop of ψ_pre_ was markedly small in invasive *B*. *javanica* saplings, showing that they were unable to extremely consume water in soil in the pot probably because of their high susceptibility in xylem conduits to soil desiccation. Among native trees, which are the drought-tolerant species, conspicuous variations in the progress of the ψ_pre_ drop were found with progressing drought-irrigation cycles (Fig 5). Midday leaf water potential (ψ_day_) measured on the last day in each drought period (i.e., just before irrigation) tended to decrease with each successive drought-irrigation cycle in all species, except in the invasive *B*. *javanica* (Fig 6). The decrease in ψ_day_ was most conspicuous in *H*. *glaber* (Fig 6A). In invasive *B*. *javanica*, ψ_day_ was maintained at high values and remained unchanged during the cycle treatments. *H*. *glaber* and *S*. *mertensiana* that showed significant decreases in ψ_day_ exhibited significant decreases in leaf osmotic water potential at full turgor (ψ_o_), indicating effective osmotic adjustment (Fig 6B).

### Leaf sensitivities of stomata to VPD

**Fig 7:**
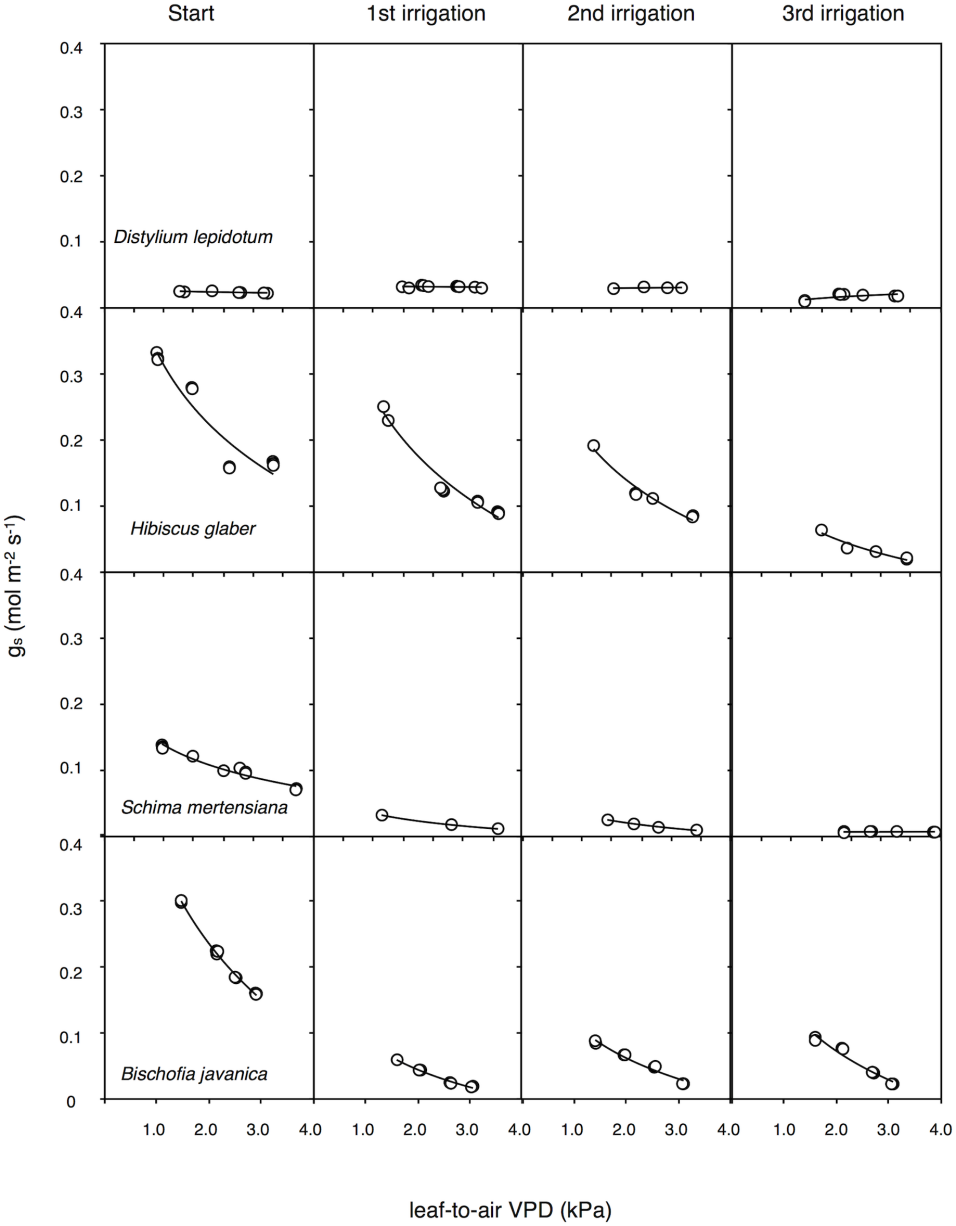
Relationships between water vapor stomatal conductance and leaf-to-air vapor pressure deficit (VPD). The measurement (Start) was conducted just before the cessation of irrigation, and the measurements (1^st^ to 3^rd^ irrigations) were conducted during the recovery period at each drought-irrigation cycle. Each panel presents a typical response in each cycle.

Large interspecific variations were found in the stomatal sensitivity to leaf-to-air VPD (*m* values), following irrigation treatments in each cycle (Fig 7, Table 1). The saplings in *B*. *javanica* and *H*. *glaber*, which exhibited recovery of embolized conduits with irrigation, had high maximum *g*_*s*_ and high sensitivity of stomata to leaf-to-air VPD at the start of the first drought-irrigation cycle (Fig 7). *B*. *javanica* showed a significant recovery of stomatal sensitivity even in the third drought-irrigation cycle (Fig 7 and Table 1). *H*. *glaber* could partly maintain stomatal regulation with the recovery of water supply, however the stomatal sensitivity decreased after each drought-irrigation cycle (but not significantly, Table 1). The stomatal sensitivities in *S*. *mertensiana* tended to be less regulated than in other species and became significantly decreased with each drought-irrigation cycle. *D*. *lepidotum* always maintained low *g*_*s*_ and stomatal sensitivity during the cycle treatments.

**Table 1:** Changes in stomatal sensitivity to leaf-to-air vapor pressure deficit under repeated drought-irrigation cycles. The values of *m* (slope index) derived from the Eq 1 and Fig 7. The slope index indicates the stomatal sensitivity to leaf-to-air vapor pressure deficit (VPD). Parenthesized numbers indicate 1 standard error of mean. *P*-values indicate the significant differences among cycles in each species (one-way ANOVA, n = 4–5).

	Start		1^st^ cycle		2^nd^ cycle		3^rd^ cycle		*p*
*D*. *lepidotum*	-0.004	(0.005)	-0.011	(0)	-0.012	na	-0.009	(0.001)	0.9439 ^ns^
*H*. *glaber*	0.25	(0.107)	0.146	(0.053)	0.125	(0.025)	0.074	(0.043)	0.4024 ^ns^
*S*. *mertensiana*	0.054	(0.021)	0.023	(0.008)	0.017	(0.007)	-0.005	(0.002)	0.0239 *
*B*. *javanica*	0.229	(0.051)	0.075	(0.028)	0.06	(0.009)	0.094	(0.031)	0.0083 **

*: *P*<0.05,

**: *P*<0.01,

^ns^: no significant difference.

### Leaf gas exchange and nitrogen contents

**Fig 8:**
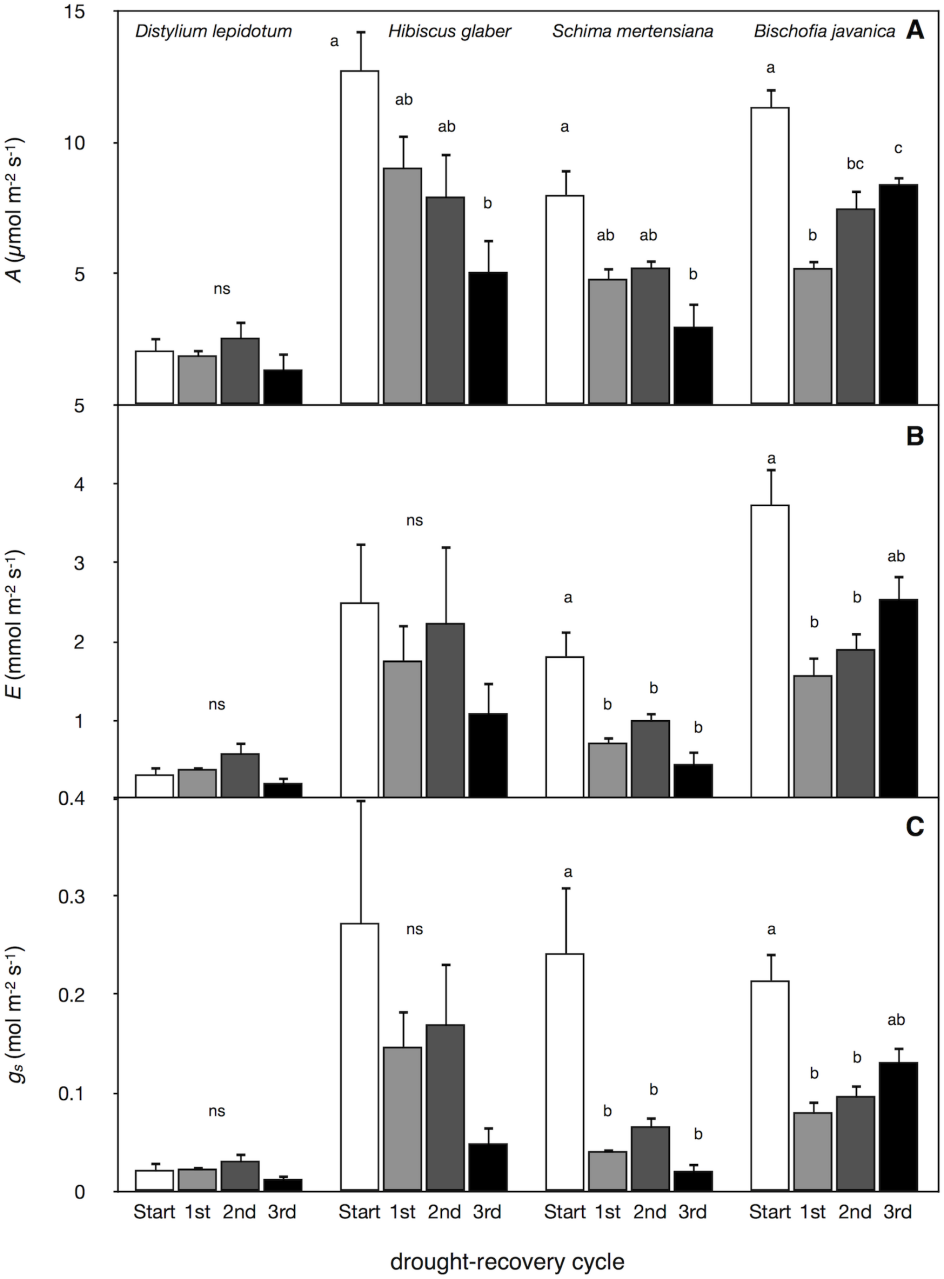
Multiple physiological traits in each irrigation period. The daily maximum values in A) the net assimilation rate (*A*), B) transpiration rate (*E*), and C) water vapor stomatal conductance (*gs*) in each irrigation period. The measurement (Start) was conducted just before the cessation of irrigation, and the measurements (1^st^ to 3^rd^) were conducted during the recovery period at each drought-irrigation cycle. Bars indicate ± 1 standard error of mean (n = 3–6). Different letters showed significant differences in each tree species (ns: no-significant differences in each species).

**Fig 9:**
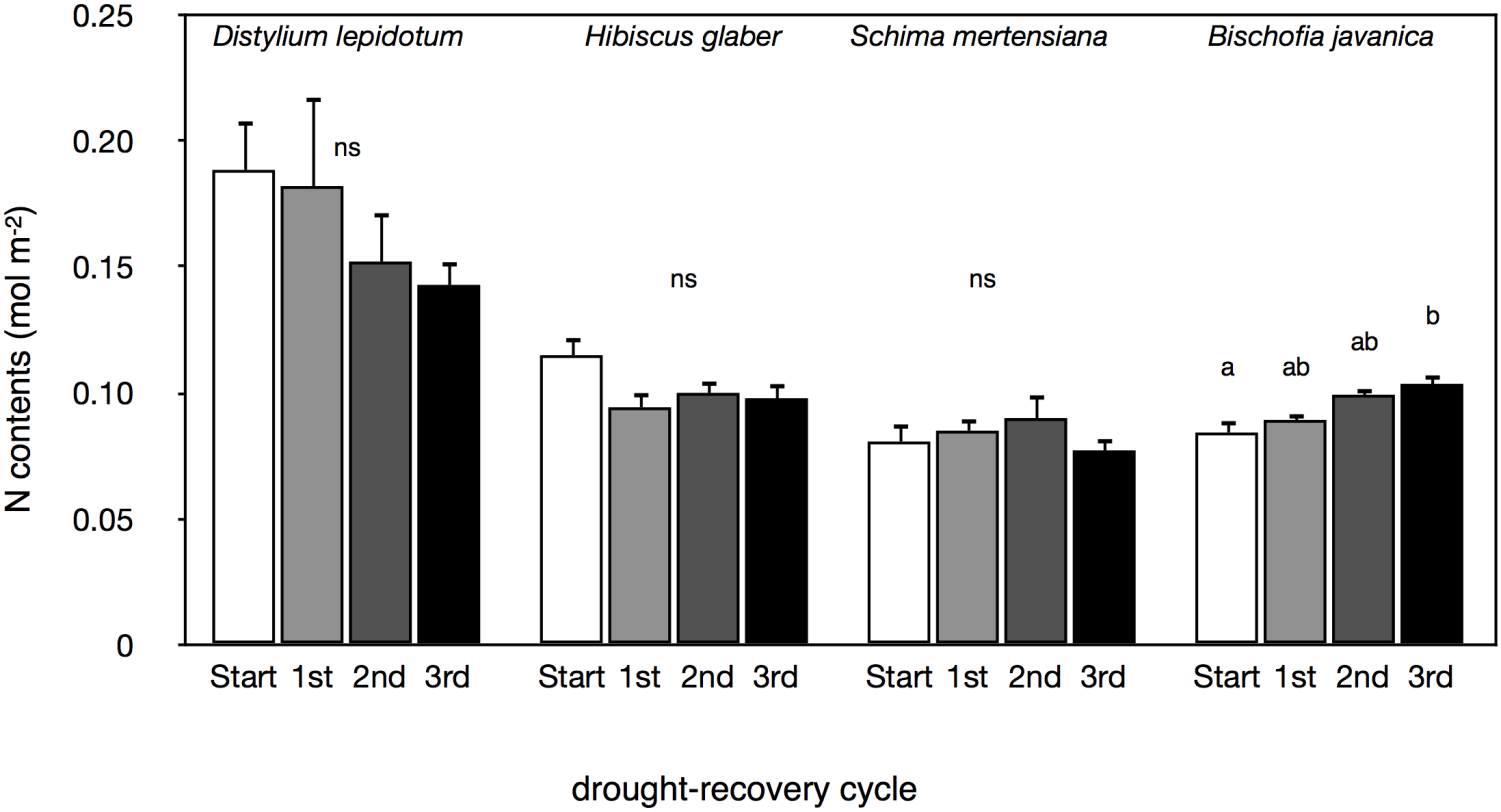
Changes in nitrogen contents under repeated drought-irrigation cycles. The values in nitrogen contents per unit leaf area in each irrigation period. The measurement (Start) was conducted just before the cessation of irrigation, and the measurements (1^st^ to 3^rd^) were conducted during the recovery period at each drought-irrigation cycle. Bars indicate ± 1 standard error of mean (n = 3–6). Different letters showed significant differences in each tree species (ns: no-significant differences in each species).

Like the measurement of stomatal sensitivity, leaf gas exchange was measured under high soil water potential following irrigation treatment in each drought cycle (Fig 8). The maximum net assimilation rate (*A*), transpiration rate (*E*), and water vapor stomatal conductance (*g*_*s*_) per unit leaf area were smallest in *D*. *lepidotum* among species and no significant variations in *A*, *E*, and *g*_*s*_ were found among the drought-irrigation cycles in *D*. *lepidotum* (Fig 8). These values in leaf gas exchange tended to decrease with the drought-irrigation cycles in *H*. *glaber* and *S*. *mertensiana* (Fig 8). The remaining canopy leaves in *B*. *javanica* showed a gradual recovery in leaf gas exchange with repeated drought-irrigation treatments (Fig 8), probably resulting from high soil water potential and conspicuous leaf shedding (Figs 1 and 5). Water used in the repeated irrigation treatments included fertilization, yet no increase in the leaf N contents per unit leaf area, which likely contributed to an increase in *A*, was found in all except the remaining leaves of invasive *B*. *javanica* (Fig 9).

## Discussion

Invasive *B*. *javanica* trees favor moist soil in the Bonin Islands. Both strong stomatal regulation (Fig 7) and conspicuous leaf shedding (Fig 1) seem to aid in the maintenance of high leaf water potential under fluctuating water regimes, i.e., the “isohydric” behavior [39]. Photosynthetic rates only recovered in *B*. *javanica*, which exhibited leaf shedding under high ψ_pre_ conditions. *B*. *javanica* has a high acclimation ability to changing light conditions [1,40] and nitrogen in soil [2]. These physiological traits have potentially facilitated the invasion of *B*. *javanica* on the Bonin Islands. Our results also suggest that *B*. *javanica* saplings presumably manage a fluctuating water supply accompanied by hydraulic segmentation of leaves [41,42] and recovery of the stomatal responses to VPD. *B*. *javanica* trees can easily conduct shoot sprouting after damaging. Following the first cycle, *B*. *javanica* saplings grew new leaves (Fig 1), assuming that they used the remaining water in the pots, and even had recovered in *A* in the remaining leaves (Fig 8). The high acclimation ability of *B*. *javanica* to the change in nitrogen supply [2] may have facilitated recovery of their productivity. In the Bonin Islands, however, *B*. *javanica* trees are mainly distributed among mesic and deep soil sites. Isohydric behavior of invasive *B*. *javanica* would normally be accompanied by lower water consumption in soil, though they may effectively use sudden water supply even when they are not under severe drought conditions. The physiological recovery of *B*. *javanica* may be limited at more xeric sites because the low water potential gradient between leaves and soil (e.g., Figs 5 and 6) may be insufficient to create a driving force to pull up water at xeric sites. Under prolonged drought without plenty precipitation, they can not sustain a high competitive advantage, resulting in the fact that the distribution of invasive *B*. *javanica* is limited in mesic/moist sites in the islands [1,40].

We found that cavitation tolerance and partial leaf shedding were species specific, and the consumption of available water was dependent on the water-use strategy, i.e., the mechanism of drought tolerance in plant species. Both *B*. *javanica* and *S*. *mertensiana* showed evidence of leaf shedding with the formation of an abscission layer during drought progression (Fig 1). Shedding of the abscission layer seemed to be involved in the progression of cavitation in the xylem during the drought period (Figs 2 and 3). Leaf shedding during the drought period may act as a hydraulic circuit breaker to conserve their hydraulic function [42] and to reduce transpiration area, which ultimately conserves water use in whole plants [19,41,43]. Neither *S*. *mertensiana* nor *H*. *glaber* fully recovered their pre-drought photosynthetic rate under full irrigation following prolonged drought (Fig 8). *S*. *mertensiana* exhibited effective osmotic adjustment in the remaining leaves (Fig 6) but showed no recovery of stomatal response (Fig 7), resulting in reduced photosynthesis activity (Fig 8). *H*. *glaber* also exhibited effective osmotic adjustment and high maximum g_s_ (Figs 6 and 8) and probably, the plants consumed as much water as possible in the pots without shedding leaves under prolonged drought, while maintaining water in their conduits for 15 days without irrigation. However, leaf shedding was adequate to conserve water under repeated water shortage. While we revealed species-specific differences in water dynamics during severe drought and irrigation, we could not determine whether the embolized conduits refilled after irrigation [44,45]. In the drought deciduous species in this study, *B*. *javanica* has a higher ratio of ray parenchymal cells in the xylem than *S*. *mertensiana* (Yazaki, unpublished data). Differences in xylem structure are associated with water conservation in xylem conduits [27,30,46–49]. These structural traits and cellular functions of the stem xylem may be related to the recovery of productivity shown only in *B*. *javanica* under the fluctuating water regime. Our results illustrate how cavitation tolerance and leaf shedding are associated during drought conditions.

Among the native trees growing along the dry ridges, *D*. *lepidotum*, which has low water potential in the field [35,50], showed the most typical water saving strategy, whereas *H*. *glaber* showed typical water consumption strategy. The lowest *g*_s_ and *A* in *D*. *lepidotum* indicate effective adaptation for avoiding cavitation formation, probably relating to the fact that the photosynthetic rates remained unchanged following the drought cycle in only *D*. *lepidotumn*. In contrast, *H*. *glaber* would have higher hydraulic efficiency than that of *D*. *lepidotumn* [35] because of the wide xylem vessels and water conservation (Figs 2–4). These traits would give *H*. *glaber* an advantage in surviving fluctuating water conditions. Because the stems of *H*. *glaber* adult trees have relatively lower wood density (0.773 g cm^-3^ in *D*. *lepidotum* and 0.600 g cm^-3^ in *H*. *glaber* [35]) and high *P*50 (unpublished data), they should favor relatively frequent rainfall. The fact that they can grow on dry ridges in shallow soil on the islands seems to support that cavitation tolerance of the main vessel (Fig 4) is important for living. A severe drought occurred in June-July of 2011 in the Bonin Islands, and during this period dieback due to dehydration was found in trees with relatively high wood density, such as *D*. *lepidotum* and *Dodonaea viscosa* (L.) *Jacquin*, favoring more xeric ridge sites with shallow soil rather than *H*. *glaber*, which have lower wood density (personal observation). It is possible that during this period, water potential dropped to a lethal level at shallow soil sites. Such a paradoxical feature was also found in forests in California in 2007, when a prolonged drought occurred [7].

Ishida et al. [34] suggested the differences in functional types with contrasting plant strategies can be attributed to functional integration among leaf carbon economy, hydraulics, and leaf longevity. Yazaki et al. [19] showed that *Trema orientalis*, native and early successional tree species in the Bonin Islands, is prone to leaf shedding under drought without any leaf osmotic adjustment ability. In terms of the cost-benefit concept, the abscission of leaves with high leaf construction cost (i.e., high leaf mass per area (LMA) and long leaf lifespan) is disadvantageous in relation to the carbon economy [51]. In this study, *S*. *mertensiana* had similar LMA (mean ± S.D is 53.2 ± 8.4 in *B*. *javanica* and that is 59.9 ± 9.1 in *S*. *mertensiana*), and they shed their leaves during the first drought period (Fig 1). However, they exhibited different water saving manner and recovery processes in spite of similar LMAs. These differences may be attributed to the balance of carbon investment among structural carbohydrates (construction cost) and non-structural carbohydrates (osmoles of leaves or living cells). We must estimate the carbon cost of water conservation accompanied by leaf segmentation and osmotic adjustment to predict species mortality under drought based on the carbon balance [8].

## Conclusions

We hypothesize that a combination of cavitation tolerance and leaf shedding underlie interspecific variation in water use and drought tolerance under fluctuating drought conditions. Invasive *B*. *javanica*, which exhibited a recovery of production from prolonged drought, may have a greater potential to expand in distribution than other native species under the frequent occurrence of drought-irrigation cycles. The fluctuation of precipitation can potentially change tree distribution even in mesic or moist sites under on going changes in precipitation in the Bonin Islands. Overall, our results suggest that environmental fluctuations are important factors for success of invasive trees. It has been reported that extreme fluctuations in dry or rainy seasons, which will result in hydraulic failure in trees, has increased in many regions in the world, as a result of global warming [52]. The hypothesized mechanisms in mortality under fluctuating water regimes will facilitate the understanding of co-existence mechanisms in many tree species at the ridge sites, and help predict changing ecosystem structure and functions of forests in the future [7,8]. More study is required to evaluate the effects of fluctuating precipitation on forest structure and dynamics under a changing environment.

## Supporting Information

S1 Dataset Dataset which is used in this study. (XLS)
